# Paclitaxel alters the expression and specific activity of deoxycytidine kinase and cytidine deaminase in non-small cell lung cancer cell lines

**DOI:** 10.1186/1756-9966-28-76

**Published:** 2009-06-06

**Authors:** Stacy S Shord, Shitalben R Patel

**Affiliations:** 1 University of Illinois at Chicago, College of Pharmacy (MC 886), Department of Pharmacy Practice, 833 South Wood Street, Room 164, Chicago, Illinois 60612, USA

## Abstract

**Background:**

We observed that paclitaxel altered the pharmacokinetic properties of gemcitabine in patients with non-small cell lung cancer (NSCLC) and limited the accumulation of gemcitabine and its metabolites in various primary and immortalized human cells. Therefore, we classified the drug-drug interaction and the effects of paclitaxel on deoxycytidine kinase (dCK) and cytidine deaminase (CDA) in three NSCLC cell lines. These enzymes are responsible for the metabolism of gemcitabine to its deaminated metabolite dFdU (80% of the parent drug) and the phosphorylated metabolites dFdCMP, dFdCDP and dFdCTP. These metabolites appear to relate to sensitivity and tolerability of gemcitabine based on previous animal and laboratory studies.

**Methods:**

Three immortalized human cells representative of the most common histological subtypes identified in patients with advanced NSCLC were exposed to the individual drugs or combinations to complete a multiple drug effect analysis. These same cell lines were exposed to vehicle-control or paclitaxel and the mRNA levels, protein expression and specific activity of dCK and CDA were compared. Comparisons were made using a two-tailed paired t-test or analysis of variance with a P value of < 0.05 considered significant.

**Results:**

The multiple drug effect analysis indicated synergy for H460, H520 and H838 cells independent of sequence. As anticipated, paclitaxel-gemcitabine increased the number of G2/M cells, whereas gemcitabine-paclitaxel increased the number of G0/G1 or S cells. Paclitaxel significantly decreased dCK and CDA mRNA levels in H460 and H520 cells (40% to 60%, P < 0.05) and lowered dCK protein (24% to 56%, P < 0.05) without affecting CDA protein. However, paclitaxel increased both dCK (10% to 50%) and CDA (75% to 153%) activity (P < 0.05). Paclitaxel caused substantial declines in the accumulation of the deaminated and phosphorylated metabolites in H520 cells (P < 0.05); the metabolites were not measurable in the remaining two cell lines. The ratio of dCK to CDA mRNA levels corresponded to the combination index (CI) estimated for sequential paclitaxel-gemcitabine.

**Conclusion:**

In summary, paclitaxel altered the mRNA levels and specific activity of dCK and CDA and these effects could be dependent on histological subtype. More cell and animal studies are needed to further characterize the relationship between mRNA levels and the overall drug-drug interaction and the potential to use histological subtype as a predictive factor in the selection of an appropriate anticancer drug regimen.

## Background

Lung cancer develops in more than 200,000 people and causes more than 160,000 deaths each year; non-small cell lung cancer (NSCLC) is the most common type of lung cancer. Cisplatin doublets remain the cornerstone of treatment[1]; however, the median overall survival remains less than one year despite multiple combinations of third generation cytotoxic drugs and novel targeted therapies. Anticancer drug regimens selected based on newly identified predictive factors may lead to an improvement in outcomes. For example, Scagliotti and colleagues[2] demonstrated that the histological subtype predicted clinical outcomes associated with different cisplatin doublets.

Gemcitabine and paclitaxel is a rationale alternative drug combination, since these anti-cancer drugs have different mechanisms of action and only partially overlapping toxicity and these drugs are among the most active anti-cancer drugs for NSCLC [3,4]. Gemcitabine is a fluorinated pyrimidine analog that causes masked chain termination and inhibits ribonucleotide reductase (RNR) [5]. It induces a G0/G1 or S phase arrest and triggers apoptosis in both hematological malignancies and solid tumors. Gemcitabine undergoes sequential intracellular phosphorylation by deoxycytidine kinase (dCK) and other nucleoside kinases to an active metabolite, difluorodeoxycytidine triphosphate (dFdCTP). The triphosphate is incorporated into DNA and inhibits DNA synthesis by stopping chain elongation. The diphosphate metabolite (dFdCDP) potentiates the incorporation of the dFdCTP into DNA by inhibiting RNR. This reduces the intracellular accumulation of deoxycytidine triphosphate (dCTP) and promotes the incorporation of dFdCTP into DNA. Reducing the intracellular accumulation of dCTP also inhibits deoxycytidine monophosphate deaminase and helps to maintain the nucleotide pool needed to form the phosphorylated metabolites. Essentially, gemcitabine potentiates its own cytotoxicity. The accumulation of the triphosphate and alterations in either dCK or RNR are associated with either sensitivity or resistance to gemcitabine in various cell lines and animal models [6-10].

Gemcitabine also undergoes intracellular and extracellular metabolism by cytidine deaminase (CDA) to purported inactive metabolite, difluorodeoxyuridine (dFdU). The deamination pathway accounts for at least 77% of the administered dose with about 5% of the parent drug gemcitabine excreted unchanged in the urine within the first six hours [11]. Reduced deamination contributes to myelosuppression based on a recent study conducted in a mouse model [12].

Paclitaxel is a natural product isolated form a pacific yew tree that induces a G2/M phase arrest by binding and stabilizing microtubules in solid tumors [13]. It is metabolized by cytochrome P450 enzymes to two potentially active metabolites. The most common toxicities include myelosuppression and peripheral neuropathy.

Clinical studies incorporating combinations of gemcitabine and paclitaxel were initiated more than 10 years ago. Many of these clinical trials indicated paclitaxel-gemcitabine provides patients with improved response rates compared to gemcitabine or paclitaxel alone, but further examination of these studies revealed that the combination provides only marginal benefit compared to each agent alone and appears inferior compared to other combinations [4,14,15]. However, this combination could prove beneficial to some patients if appropriately selected based on histological subtype or molecular markers. Our data supports that paclitaxel reduces the volume of distribution and systemic clearance of gemcitabine and paclitaxel limits the uptake and metabolism of the gemcitabine in primary and immortalized human cells [16,17]. In the present study, we further investigated this combination and the effects of paclitaxel on the mRNA levels, protein expression and specific activity of dCK and CDA based on our observations that paclitaxel reduces the systemic clearance in humans and the accumulation of the metabolites in the laboratory. For this purpose, we treated three separate immortalized human NSCLC cell lines obtained from patients diagnosed with advanced disease that represent the more common histological subtypes.

## Methods

### Chemicals

Gemcitabine (Gemzar^®^; 2',2'-difluoro- 2'-deoxycytidine; dFdC) was a generous gift from Eli Lilly and Company (Indianapolis, IN) and dissolved in sterile distilled water. Paclitaxel was purchased from Sigma-Aldrich Company (St. Louis, MO) and dissolved in 0.1% acetic acid in methanol. Radiolabeled chlorodeoxyadenosine (8-^3^H-CdA, 7.8 Ci/mmol) was purchased from Moravek (Brea, CA). All other chemicals were of analytical grade.

### Cell culture

The NSC large cell lung carcinoma H520 cell line (mutant-p53) was provided by Dr. William T. Beck (University of Illinois, Chicago, Illinois, USA). The NSC H460 squamous carcinoma cell line (wild-type p53) and H838 adenocarcinoma cell line (wild-type p53) were obtained from the American Type Culture Collection (Manassas, Virginia, USA). The cells were grown in monolayers and maintained in exponential growth in RPMI-1640 medium containing 2 mM L-glutamine supplemented with 10% fetal bovine serum (FBS) and 1% penicillin (10,000 U penicllin per ml)-streptomycin (10 mg of streptomycin per ml) at 37°C at 5% CO_2_. The medium was further supplemented with insulin (Gibco Life Technologies, Grand Island, New York, USA) for H520 cells.

### Growth inhibition assay

Growth inhibition was determined using a dye exclusion assay with trypan blue staining followed by a cell count using a hemocytometer [18]. Briefly, ~3.5 × 10^5 ^cells were seeded in duplicate in 6-well flat bottom plates. After 24 hours, the cells were treated with vehicle-control, gemcitabine (ranged from 1 to 15,000 nM) or paclitaxel (ranged from 1 to 3,000 nM) for 24 hours. The fraction of affected cells and unaffected cells for the individual drugs was calculated compared to cells exposed to vehicle-control. The IC_50 _values were determined using linear regression analysis with the aide of CalcuSyn software (v. 2, Biosoft, Cambridge, UK).

A multiple drug effect analysis was completed to predict the likely drug-drug interaction based on the principles of Chou and Talalay [19]. The combination index (CI) for each fraction affected was simulated and for the final evaluation, the averaged CI at 0.50, 0.75, 0.90 and 0.95 fraction affected was determined [20]. Briefly, ~1 × 10^6 ^cells were seeded in duplicate in 60 mm dishes. After 24 hours, the cells were treated with sequential gemcitabine → paclitaxel and paclitaxel → gemcitabine at five different concentrations of a constant ratio based on the ratio of the observed IC_50 _values of each cell line. The cells were exposed to each drug for 24 hours; the medium containing the first drug was removed, the cells were washed with phosphate buffered saline, then medium containing the second drug was added to the cells. The total culture time was 72 hours. A CI < 0.3, 0.3–0.7, 0.7–0.9, 0.9–1.1, 1.1–1.45, 1.45–3.3 and >3.3 indicates highly synergistic, synergistic, moderate to slight synergistic, nearly additive, slight to moderate antagonistic, antagonistic; strong antagonistic, respectively (CalcuSyn software, v. 2, Biosoft, Cambridge, UK).

### Flow cytometry

Flow cytometric measurements were performed after staining the cellular DNA content with propidium iodide to determine the cell cycle distribution and apoptosis following treatment with sequential gemcitabine → paclitaxel or paclitaxel → gemcitabine. Briefly, ~1 × 10^6 ^cells were plated in 60 mm dishes and allowed to attach overnight. After treatment with sequential gemcitabine → paclitaxel or paclitaxel → gemcitabine as described for the determination of the CI, the cells were harvested and suspended in a propidium iodide solution (Sigma-Aldrich Co.) as described previously [21] and filtered in 5 ml round bottom tube with cell-strainer cap (BD Falcon). The cell cycle analysis was performed on a Beckman-Coulter EPICS Elite ESP flow cytometer (Hialeah, Florida, USA) using the Multicycle AV program (v. 3, Phoenix Flow Systems, San Diego, Calfornia, USA).

### dCK and CDA enzyme specific activity

The effect of paclitaxel on dCK and CDA enzyme specific activity was measured after exposing ~20–30 × 10^6 ^cells (seeded in duplicate in 100 mm dishes) to either vehicle-control or paclitaxel at the observed IC_50 _value for 24 hours. Cells were manually harvested and counted. Total protein was quantified using BCA protein kit (Pierce Biotechnology, Rockford, Illinois, USA)

dCK activity was analyzed using radiolabeled chlorodeoxyadenosine (CdA) as previously described [22,23]. Briefly, the crude cellular extract was suspended in Tris-HCl buffer and mixed with CdA 256.5 μM plus [8-^3^H]-CdA (128 μM, specific activity 0.19 μCi/nmol) as substrate. The enzymatic reaction was incubated for 1 hour at 37°C. Enzyme activities were expressed as nmol product formed per hour per mg protein or 10^6 ^cells.

The CDA activity was measured using a spectrophotometric method as described by Dr. Vincenzetti [24]. The crude cellular extract was suspended in a Tris-HCl buffer and freeze-thawed rapidly three times. The extract was subsequently centrifuged for 15 minutes at 12,000 *g *and the resulting supernatant was suspended in the Tris-HCl buffer. The enzymatic reaction was performed in a 96 well UV-Vis transparent plate (BD Falcon) and initiated with the addition of the substrate cytidine (167 μM). The mixture was incubated at 37°C and the change in absorbance at 282 nm was measured for 10 minutes using a Synergy HT multidetection microplate reader (Biotek, Winooski, Vermont, USA). Enzyme activities were expressed as mmol substrate consumed per minute per mg protein or 10^6 ^cells.

### Gene expression

Total RNA and protein was extracted form cells exposed to vehicle-control or paclitaxel at varying concentrations for 24 hours using the PARIS™ kit (Ambion, Austin, Texas, USA) according to manufacturer's instructions. Total RNA was treated with TURBO DNA free (Ambion) to remove DNA contamination and the concentration was measured at 260 nm. The total RNA was reverse transcribed using random primers and the High Capacity cDNA reverse transcription kit (Applied Biosystems) per the manufacturer's product information.

The human hypoxanthine phosphoribosyltransferase *(HPRT) *gene was selected as an endogenous control after assessing the gene expression of 11 potential controls using the TaqMan human endogenous control plate (Applied Biosystems). *HPRT *produced ΔC_T _values that deviated little from zero, indicating relative to other candidate controls, that the expression of *HPRT *remains relatively consistent across the samples tested regardless of type of cells or treatment. Primers and probes for the *dCK *and *CDA *were from Applied Biosystems Assay on-Demand Gene expression products. The cDNA was amplified by quantitative real-time PCR in triplicate using the following thermal profile: an initial incubation at 50°C for 5 minutes, followed by 40 cycles of denaturation at 95°C for 15 seconds followed by annealing and extension at 60° for 1 minute with the Applied Biosystems 7900 HT sequence detection system. The quantitation of gene expression was performed relative to the calibrator (vehicle-control cells) using the ΔΔC_T _calculation for dCK and the relative standard curve calculation for CDA. A validation experiment was performed that demonstrated the efficiencies were 0.08 for dCK and 1.1 for CDA. To use the ΔΔC_T _calculation, the efficiencies should be less than 0.1.

### Western blot

Total protein was separated on a 12% SDS-polyacrylamide gel for dCK or a 14% SDS-polyacrylamide gel for CDA and transferred to a polyvinylidene diflouride (PVDF) membrane [25,26]. The membrane was probed with the either dCK-pep antibody (obtained from Dr. Hatzis) at a 1:4,000 dilution or CDA antiserum (obtained from Dr. Momparler) at a 1:175 dilution followed by incubation with horseradish peroxidase-conjugated anti-rabbit IgG (Pierce, Rockford, Illinois, USA). The membrane was also probed with β-actin (Sigma-Aldrich Co) at 1:12,000 dilution, followed by incubation with horseradish peroxidase-conjugated anti-mouse IgG (Calbiochem, San Diego, California, USA) antibody as an endogenous control. Immuncomplexes were visualized by SuperSignal West Pico chemiluminescent substrate kit (Pierce, Rockford, IL) and the band density was semi-quantitated using ImageJ (v. 1.38×, ) software.

### Analysis of gemcitabine, difluorodeoxyuridine and the phosphorylated metabolites

The cells were treated with vehicle-control or paclitaxel at the observed IC-50 value for 24 hours followed by gemcitabine at the observed IC-50 value for 24 hours. The cell medium and pellet were manually harvested and stored at -80°C until analysis. The phosphorylated metabolites were analyzed by Dr. Hilde Rosing within the Department of Pharmacy and Pharmacology at the Netherlands Cancer Institute/Slotervaart Hospital in Amsterdam, Netherlands using their previously described LC-MS method [27]. The lower limit of quantitation was 26.8 nM for the monophosphate, 27.0 nM for the diphosphate and 2.53 nM for the triphosphate.

Gemcitabine and its deaminated metabolite dFdU were analyzed in our laboratory using our previously published method with hexanes used to wash the culture medium [28]. The lower limit of quantitation was 0.25 μM for both gemcitabine and dFdU.

### Statistical analysis

All results are expressed as the mean ± the standard deviation of three independent experiments conducted in at least triplicate. Statistical significance was determined by a two sided paired *t *test or analysis of variance and the level of significance was set at *P *< 0.05 a priori. A correlation analysis was conducted to determine the relationship between the ratio of dCK to CDA mRNA levels and combination index.

## Results

### Effects of gemcitabine and paclitaxel on cell viability

Table 1 summarizes the sensitivity of H460, H520 and H838 cell lines to gemcitabine and paclitaxel. H460 cells were the most sensitive to gemcitabine and H838 cells were the most sensitive to paclitaxel. From these data, the ratio of the observed IC-50 values of gemcitabine to paclitaxel was determined and used to perform the multiple drug effect analysis.

**Table 1:** Sensitivity of solid tumor cells lines to gemcitabine and paclitaxel

Cell line/Exposure	H460	H520	H838
IC-50 Gemcitabine (nM)			
24 h	6.7	1541.1	72.8

IC-50 Paclitaxel (nM)			
24 h	178.0	241.6	7.2

The IC-50 is defined as the concentration that causes 50% inhibition of cell growth after exposure to either gemcitabine 24 h or paclitaxel 24 h. Growth inhibition was determined using a direct cell count and the fraction affected was averaged from three independent experiments with six replicates to calculate the IC-50 using CalcuSyn (v 2.0, Biosoft).

**Figure 1:**
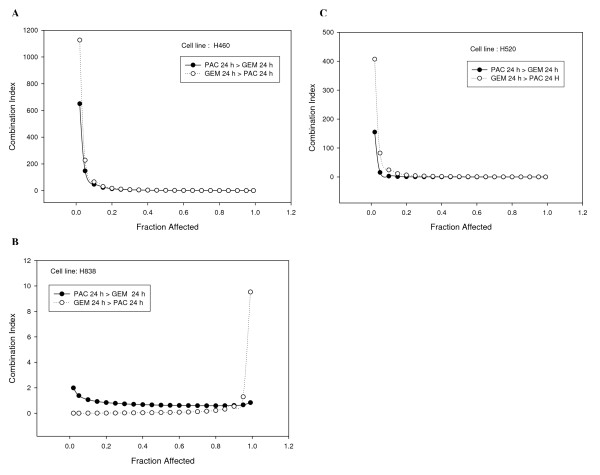
**Combination index values and fraction of cells affected for three non-small cell lung cancer cell lines exposed to paclitaxel followed by gemcitabine or gemcitabine followed by paclitaxel at 24 hours interval with a total culture time of 48 h**. (a) H460, squamous cell carcinoma; (b) H838, adenocarcinoma carcinoma and (c) H520, large cell carcinoma.

Table 2 summarizes the average CI for these cell lines for 0.50, 0.75, 0.90 and 0.95 fraction affected and Figure 1 illustrates the CI vs. the fraction of affected cells exposed to sequential paclitaxel-gemcitabine or gemcitabine-paclitaxel. The interaction was classified as synergistic for all three cell lines independent of sequence based on the average CI, but the individual curves suggest that predicted interaction may be dependent on the drug concentration. For example, the CI predicts additivity or antagonism as the fraction affected approaches 100% in H460 cells.

**Table 2:** Combination index of solid tumor cell lines for gemcitabine and paclitaxel

Exposure/Cell line	H460	H520	H838
PAC 24 h > GEM 24 h	0.58 ± 0.84	0.006 ± 0.010	0.63 ± 0.03
Predicted Interaction	Synergistic	Highly Synergistic	Synergistic
GEM 24 h > PAC 24 h	0.60 ± 0.91	0.34 ± 0.41	0.50 ± 0.57
Predicted Interaction	Synergistic	Synergistic	Synergistic

Mean (± standard deviation) CI values after exposure to paclitaxel for 24 hours followed by gemcitabine for 24 hours or gemcitabine for 24 hours followed by paclitaxel 24 hours. The mean CI values represent the average of the CI at the fraction affected of 0.50, 0.75, 0.90 and 0.95. Cells were seeded in 6-well flat bottom plates in duplicate at 5 separate concentrations of constant ratio based on the ratio of the observed IC-50 values. Three independent counts were conducted for each well with a total of six replicates and the CI was determined using an algebraic estimation algorithm with the aide of CalcuSyn (v 2.0, Biosoft).

Comparing the fraction affected indicates a sequence dependent effect in two of the three cell lines (H460, H838); the sequence gemcitabine-paclitaxel was favored in these two cell lines compared to the sequence paclitaxel-gemcitabine (paclitaxel-gemcitabine vs. gemcitabine-paclitaxel, P < 0.05). However, the percentage of apoptotic cells largely favors sequential paclitaxel-gemcitabine with significantly more apoptosis found in H838 cells (P < 0.01).

### Effects of gemcitabine and paclitaxel on cell cycle distribution

Flow cytometric measurements were completed to compare the effects of sequential paclitaxel-gemcitabine and gemcitabine-paclitaxel on the cell cycle distribution. Table 2 summarizes the effects of gemcitabine and paclitaxel on cell cycle distribution. These cells were exposed to sequential gemcitabine-paclitaxel or the reverse sequence. As anticipated, paclitaxel-gemcitabine produced a sequence dependent increase in the number of G2/M cells as noted in H520 cells (paclitaxel-gemcitabine vs. gemcitabine-paclitaxel, P < 0.05) and gemcitabine-paclitaxel produced an increase in the number of G0/G1 cells as noted in H520 cells (P < 0.05).

### Effects of paclitaxel on gene expression, protein and activity of dCK

**Figure 2:**
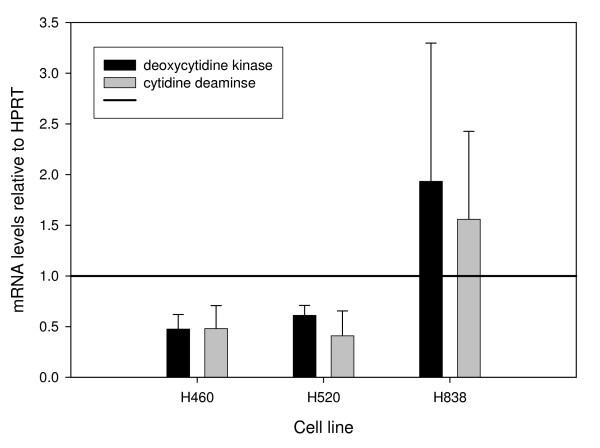
**Effects of paclitaxel on *dCK *and *CDA***. Mean (± standard deviation) relative mRNA levels of *dCK *and *CDA *to *HPRT *in paclitaxel treated cells compared to the mRNA levels of *dCK *and *CDA *to *HPRT *in vehicle control treated cells (set to the value of 1) from three independent experiments. The cells were treated with paclitaxel at the observed IC-50 value for 24 hours.

The effects of paclitaxel on dCK mRNA levels were measured by quantitative RT-PCR using ΔΔC_T _method (Figure 2). The mRNA expression was significantly decreased in paclitaxel vs. vehicle-control treated H460 (52%, P < 0.05) and H520 (39%, P < 0.05) cells. The mRNA expression was relatively unchanged in the H838 cells.

**Figure 3:**
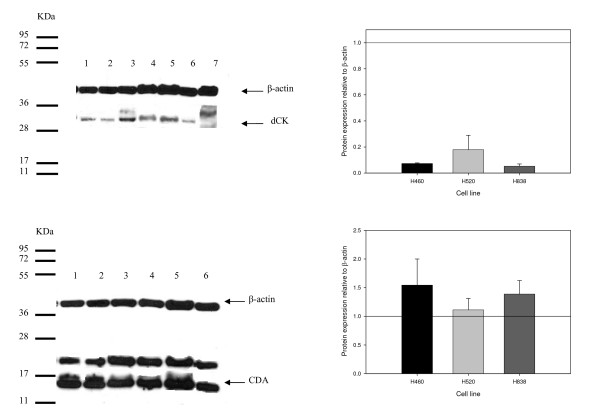
**dCK and CDA protein expression in non-small cell lung cancer cell lines**. (a) A representative Western immunoblot of crude cellular extracts from H460 (lane 1,2), H520 (lane 3,4), H838 (lane 5,6) and AG6000 (A2780 variant without dCK, lane 7). The odd lanes were treated with vehicle-control and the even lanes were treated with paclitaxel at the observed IC_50 _value for 24 hours. (b) The mean (± standard deviation) relative protein levels of dCK to β-actin after exposure to paclitaxel at the observed IC-50 value for 24 hours compared to vehicle-control (set to the value of 1) from three independent experiments. (c) A representative Western immunoblot of crude cellular extracts from H460 (lane 1,2), H520 (lane 3,4), and H838 (lane 5,6). The odd lanes were treated with vehicle-control and the even lanes were treated with paclitaxel at the observed IC_50 _values for 24 hours. (d) The mean (± standard deviation) relative protein levels of CDA to β-actin treated with paclitaxel at the observed IC-50 value for 24 hours compared to relative protein levels of CDA to β-actin treated with vehicle-control (set to the value of 1) from three independent experiments.

The effects of paclitaxel on dCK protein were measured by Western immunoblot analysis (Figure 3). The protein expression decreased by 24 to 56% in all cell lines, but the decrease was only statistically significantly lower in paclitaxel-treated H460 cells compared to vehicle-control treated cells (P < 0.05).

The enzyme specific activities of dCK are summarized in Table 3. The cells were exposed to vehicle-control or paclitaxel at the observed IC-50 value determined in the specific cell line. Basal dCK activity was highest in H838 cells and lowest in H460 cells. The mean activity increased 10 to 50% in all of the cell lines, but the increase in activity was only statistically significantly higher in H460 and H520 cells treated with paclitaxel compared to vehicle-control (P < 0.05).

**Table 3:** Effects of paclitaxel on deoxycytdine kinase and cytidine deaminase activity in solid tumor cell lines

Exposure/Cell line	H460	H520	H838
Control			
%G0 + G1	66 ± 1.2	62 ± 2.1	80 ± 7.5
%G2 + M	8.0 ± 1.4	13.2 ± 1.0	4.8 ± 2.4
%S	26 ± 1.7	25 ± 1.3	15 ± 5.1
% Apoptosis	7.5 ± 1.7	3.2 ± 0.6	9.7 ± 7.2

PAC 24 h > GEM 24 h			
%G0 + G1	17 ± 11	36 ± 6.4	23 ± 6.0
%G2 + M	25 ± 7.8	44 ± 6.4^a^	15 ± 4.7
%S	58 ± 3.2	20 ± 2.3	41 ± 1.0
% Apoptosis	8.6 ± 5.1	2.1 ± 1.4	4.6 ± 1.0

GEM 24 h > PAC 24 h			
%G0 + G1	13 ± 6.0	62 ± 4.9^a^	23 ± 10.3
%G2 + M	30 ± 1.7	9.7 ± 1.6	9.8 ± 8.0
%S	56 ± 7.7	28.8 ± 3.5	43 ± 1.6
% Apoptosis	7.0 ± 4.9	3.4 ± 2.2	0.87 ± 0.05^a^

Mean (± standard deviation) percentage of cells in each phase of the cell cycle after exposure to vehicle control or sequential paclitaxel → gemcitabine or gemcitabine → paclitaxel at 24 hours intervals. The mean represents three independent experiments with each experiment conducted in at least triplicate.

^a ^P < 0.05, paclitaxel vs. vehicle-control treated cells

### Effects of paclitaxel on gene expression, protein and activity of CDA

The effects of paclitaxel on CDA mRNA levels were measured by quantitative RT-PCR using the relative standard curve method (Figure 2). As measured for dCK mRNA levels, the mRNA levels was statistically significantly decreased in H460 (52%, P < 0.05) and H520 (59%, P < 0.05) cells treated with paclitaxel compared to vehicle-control. The mRNA levels were relatively unchanged in the H838 cells.

The effects of paclitaxel on CDA protein were measured by Western immunoblot analysis. The protein expression was unchanged in all three cell lines after treatment with paclitaxel at the observed IC-50 value for 24 hours compared to vehicle-control (Figure 3).

The activities of CDA are summarized in Table 4. The cells were exposed to vehicle-control or paclitaxel at the observed IC-50 value determined in the specific cell line. Basal CDA activity was highest in H520 cells and lowest in H838 cells. The mean activity increased in all three cell lines 75% to 153%, but the increase in activity was only statistically significantly higher in H520 cells treated with paclitaxel compared to cells treated with vehicle-control treated cells (P < 0.001).

**Table 4:** The effects of paclitaxel on deoxycytdine kinase and cytidine deaminase activity and gene expression in solid tumor cell lines

Cell line	H460	H520	H838
Deoxycytidine kinase (dCK)			
Control	0.46 ± 0.12	1.23 ± 0.12	2.44 ± 1.56
Paclitaxel	0.69 ± 0.14	1.67 ± 0.25	2.60 ± 0.46
Fold change	1.5	1.4^a^	1.1
Cytidine deaminase (CDA)			
Control	11.8 ± 3.4	18.2 ± 10.5	4.1 ± 2.1
Paclitaxel	27.0 ± 16.1	31.9 ± 11.1	10.4 ± 6.8
Fold change	2.3	1.8^a^	2.5

Mean (± standard deviation) of the activity of deoxycytidine kinase (nmol per hour per 10^6 ^cells) or cytidine deaminase (pmol per minute per 10^6 ^cells) after exposure to vehicle-control or paclitaxel for 24 hours. The mean represents three independent experiments with each experiment conducted in at least triplicate. The fold change represents the mean activity after exposure to paclitaxel divided by the mean activity after exposure to vehicle-control.

^a ^P < 0.05 or P < 0.001, paclitaxel vs control-treated cells

### Effects of paclitaxel on the accumulation of the phosphorylated and deaminated metabolites

**Figure 4:**
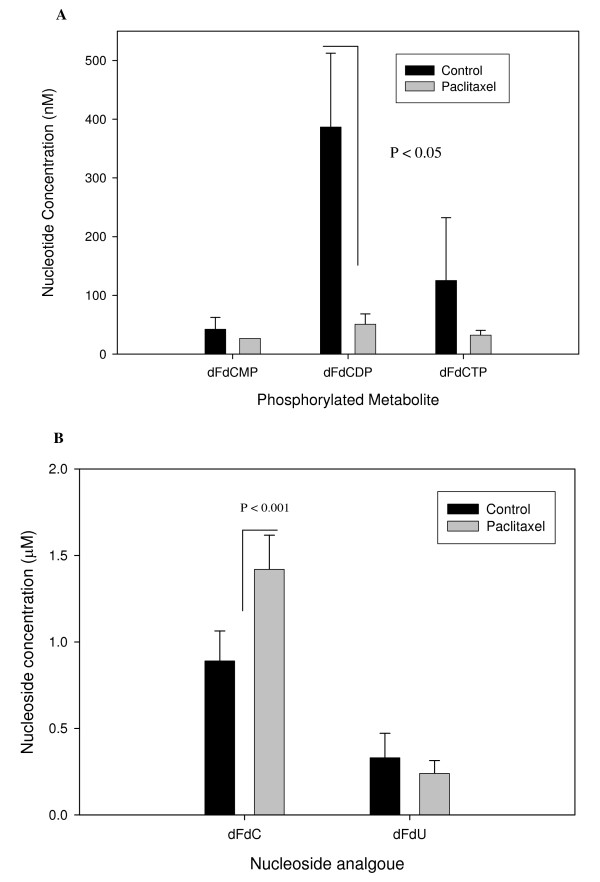
**The effect of paclitaxel on the accumulation of gemcitabine, diflourodeoxyuridine (dFdU) and the phosphorylated metabolites of gemcitabine**. The cells were treated with vehicle-control or paclitaxel at the observed IC-50 value for 24 hours followed by gemcitabine at the observed IC-50 value for 24 hours. The cell medium was collected and the cells manually harvested by a cell scraper; the medium and cells were stored at -80°C until analysis. Gemcitabine and its metabolites were below the limits of quantitation in two of the three cell lines. The accumulation of phosphorylated metabolites within the cells was measurable in (a) H520 cells and the accumulation of gemcitabine and dFdU in the medium were measurable in (b) H520 cells. The diphosphate levels were significantly lower in paclitaxel treated cells compared to vehicle-control treated cells (P < 0.05). Gemcitabine levels were significantly higher in paclitaxel treated cells compared to vehicle-control treated cells (P < 0.001).

The deaminated and phosphorylated metabolites were measurable in only the H520 cells within the medium and the cellular pellet, respectively (Figure 4). The accumulation of these metabolites was substantially decreased by paclitaxel in this cell line. The accumulation of the diphosphate exceeded the accumulation of the mono- and triphosphate. The triphosphate decreased by about 75%, the diphosphate decreased by about 87% (paclitaxel vs. vehicle control, P < 0.05) and the monophosphate decreased by about 37% in the H520 cells. The accumulation of the deaminated metabolite also decreased in the H520 cells (27%). Consistent with these substantial changes found in the metabolite accumulation (both the phosphorylated and deaminated metabolites), gemcitabine increased by 60% (P < 0.001) in paclitaxel-treated H520 cells vs. vehicle-control treated cells. This cell line was least sensitive (as noted by the IC-50 values) to gemcitabine and therefore, were treated with higher concentrations of gemcitabine which resulted in metabolite concentrations that exceeded the limits of quantitation.

### Relation between deoxycytidine kinase, cytidine deaminase and cell growth

A relationship between the ratio of the mRNA levels of dCK to CDA and the CI estimated for the sequential paclitaxel → gemcitabine was observed. The cells were treated with gemcitabine and paclitaxel at the observed IC-50 values of each drug at 24 hour intervals for a total culture time of 72 hours as described above. A CI < 1 was observed in H838 and H520 cells with a ratio of dCK to CDA > 1 and a CI = 1 was observed in H460 cells with a ratio of dCK to CDA = 1. Furthermore, the ratio of the mRNA levels strongly correlated with the combination index when examining an expanded concentration range in H838 cells (*r *= 0.90, p < 0.05). This observed relationship indicates that dCK mRNA levels are higher compared to CDA mRNA levels in cells in which the CI predicts synergism. A relationship between dCK activity or expression, CDA activity or expression, or other ratios with the CI were not observed.

## Discussion

We previously identified a possible drug-drug interaction between gemcitabine and paclitaxel; paclitaxel reduced the volume of distribution and systemic clearance of gemcitabine in humans and decreased the transport and accumulation of gemcitabine and its metabolites in primary and immortalized cells. These data appear to suggest that paclitaxel compromises the metabolism and transport of gemcitabine [16,17]. In this study, we found that paclitaxel-gemcitabine demonstrates synergy in three immortalized cells lines when the individual drug concentrations exceed the IC50 values of each individual drug, although paclitaxel substantially reduced the mRNA levels of dCK and CDA, the two enzymes responsible for the metabolism of gemcitabine to its key metabolites. We focused on the effect of paclitaxel on the metabolism of gemcitabine at this time based on previous data that indicate dCK activity corresponds to the sensitivity of murine tumors and human tumor xenografts to gemcitabine and CDA activity corresponds to myelosuppression in children [8,12].

We selected three solid tumor cell lines representing the most common histologies in patients diagnosed with advanced NSCLC; these immortalized cell lines were acquired from patients with advanced disease (H460, squamous cell carcinoma; H520, large cell carcinoma; and H838, adenocarcinoma). The multiple drug effect analysis indicates this interaction is largely independent of culture conditions or sequence; but a sequence dependent effect was noted regarding the fraction of affected cells with the gemcitabine-paclitaxel sequence favored in two of the three cell lines (H460, H838). Our results for the H460 cells compare well with a previous report in which the CI for sequential paclitaxel-gemcitabine and gemcitabine-paclitaxel was reported for similar exposure [20]. Although our data supports administering gemcitabine before paclitaxel based on the fraction affected, the percentage of apoptotic cells largely favors paclitaxel before gemcitabine consistent with the current administration of this combination to patients with advanced breast, lung or ovarian cancers. Dr. Kroep similarly concluded that sequential paclitaxel-gemcitabine was favored based on an increase in apoptosis compared to the reverse sequence [20]. As anticipated, paclitaxel increased the number of G2/M cells and gemcitabine increased the number of G0/G1 or S cells. A relationship between cell cycle distribution and the CI was not observed.

Only one other study explored possible effects of paclitaxel on dCK, but no other studies have described the effects of paclitaxel on CDA [20]. Kroep et al[20] reported that paclitaxel increased the accumulation of the triphosphorylated metabolite in H460 cells, but that dCK activity was not changed. Our findings indicate that paclitaxel increased dCK activity in all three cells lines including H460 cells and that the changes were only statistically significantly higher in the H520 cells. However, the mRNA levels were significantly reduced in two cells lines, H460 and H520, with relatively substantial decreases in protein. It is unclear why the reported outcomes are different in these studies, but the differences may be dependent on allosteric regulation governed by differences in substrate concentrations of dCTP. Additional data in breast and ovarian adenocarcinoma cells lines from our laboratory also show no change in mRNA levels and an increase in specific activity; these data are comparable to the changes observed in the lung adenocarcinoma cell line.

We also indicate that paclitaxel caused similar changes in the expression and activity of CDA. Paclitaxel substantially reduced mRNA levels in the same two cells lines in which paclitaxel decreased mRNA levels of dCK. Furthermore, CDA protein expression appears relatively unchanged by paclitaxel, but specific activity appears substantially increased. We also observed similar changes in CDA mRNA, protein and activity in two additional adenocarcinoma cell lines (breast and ovarian). We believe that our data collectively indicates that these changes may be dependent on the histological subtype, since we only observed changes in large cell and squamous cell carcinoma, and not adenocarcinoma cell lines. These experiments will need to be repeated in additional cell lines representative of these histologies to confirm our findings.

The accumulation of gemcitabine and its metabolites were only measurable in H520 cells. Most likely, it is because this cell line was least sensitive to gemcitabine (as noted by higher IC50 values) and therefore, the accumulation of these metabolites exceeded the lower limits of quantitation of the assay. Of interest, this cell expresses mutant p53, whereas the remaining two cell lines express wild-type p53. The noted differences in sensitivity to gemcitabine could be explained, in part, by p53 expression, since gemcitabine inhibits apoptosis dependent on p53 status [29]. Furthermore, the changes in the metabolite accumulation in H520 cells appears to reflect changes in dCK and CDA mRNA levels in these cell lines and further supports our findings that the CI corresponds to the ratio of dCK to CDA mRNA levels. The ratio of dCK to CDA mRNA levels could be a useful maker of response in humans. Of note, we observed that the accumulation of gemcitabine and its phosphorylated and deaminated metabolites were unchanged in an ovarian adenocarcinoma cell line; the lack of change in the accumulation of the parent drug and the metabolites in this cell line are consistent with the lack of changes in mRNA levels. This cell line also expresses mutant p53 and demonstrated IC-50 values similat to the IC-50 values of the H520 cell line [30]. Lastly, the accumulation of the diphosphate exceeded the accumulation of the triphosphate in the H520 cells treated with vehicle-control followed by gemcitabine. The triphosphate has been identified as the dominant metabolite. We used lower concentrations than those shown to maximize the accumulation of the triphosphate and harvested the cells and medium after the time of the maximal accumulation of the triphosphate and we believe these differences may explain, in part, why the diphosphate was the dominant metabolite in this cell line [31].

More studies are needed to examine the relationship of this ratio of mRNA with the CI in cell models and with response rates and survival in animal models before examining the clinical utility of this marker. Experimental conditions, including drug concentrations, treatment duration and cofactors, can sometimes limit the translation of laboratory findings to humans. But we demonstrated similar outcomes in the laboratory when the cells were treated with shorter durations comparable to the length of infusions in human and higher concentrations that can be easily achieved in the plasma of humans after a standard dose (data not shown). Therefore, we believe it is important to continue characterizing the effects of paclitaxel on the expression and activity of these proteins and determine how these modifications impact the pharmacokinetic properties and clinical outcomes in an animal model.

In summary, paclitaxel appears to modulate two key enzymes involved in the metabolism of cytidine analogues, including gemcitabine, and plays an integral role in the salvage of pyrimidine analogues. The effects on mRNA levels may be dependent on histological subtype (i.e. the effects were only noted in large cell and squamous cell carcinomas, not adenocarcinomas), but the studies need to be repeated in additional cell lines representative of the three distinct histologies. The changes in enzyme activity, in light of decreased or minimal changes in gene or protein expression, appear contradictory and could be dependent on experimental conditions (such as treatment duration, cofactors, etc), but it is possible to increase activity of these enzymes with minimal changes in protein concentrations by altering post-translational modifications (i.e. increasing nuclear localization). Of note, we obtained comparable results when exposing the cells to shorter duration (1–3 hours) or clinically achievable concentrations (3 to 15 μM) suggesting that these findings are likely independent of the experimental conditions [30]. At this time, changes in mRNA levels appear to be the predominant effects, since the ratio of dCK to CDA mRNA levels corresponding to the CI, a mathematical model commonly uses to conduct a multiple drug effect analysis, and the changes in accumulation of the deaminated and phosphorylated metabolites are in concert with the changes in mRNA levels.

## Abbreviations

CDA: cytidine deaminase; dCK: deoxycytidine kinase; dCTP: deoxycytidine triphosphate; dFdCDP: diflourodeoxycytidine diphosphate; dFdCTP: diflourodeoxycytidine triphosphate; NSCLC: non-small cell lung cancer; HPRT: human hypoxanthine phosphoribosyltransferase; RNR: ribonucleotide reductase.

## Competing interests

The authors declare that they have no competing interests.

## Authors' contributions

SS prepared the funding application that secured funding for the project; completed statistical analysis and prepared manuscript for peer-review.

SP developed and validated all assays outlined in the methods section with the exception of the liquid chromatography, completed all data analysis and helped prepared the manuscript for peer-review.
